# Philadelphia Medical Society

**Published:** 1842-01-08

**Authors:** 


### PROCEEDINGS OF SOCIETIES.

### PHILADELPHIA MEDICAL SOCIETY.

*Session of January 1st*, 1842. Dr. Reynell Coates in the Chair. Annual election. The following officers were chosen for the ensuing year:

*President*—Thomas Harris, M. D.

*Vice Presidents*—George B. Wood, M.D., Robert M. Huston, M.D.

*Treasurer*—John M. Brewer, M. D.

*Corresponding Secretaries*—W. Poyntell Johnston, M. D., B. Hornor Coates, M. D.

*Senior Recording Secretary*—J. M. Wallace, M. D.

*Orator*—Reynell Coates, M. D.

*Librarian*—J. F. White, M. D.

*Curator*—Joseph Peace, M. D.

